# Non-canonical Interaction Between O-Linked *N*-Acetylglucosamine Transferase and miR-146a-5p Aggravates High Glucose-Induced Endothelial Inflammation

**DOI:** 10.3389/fphys.2020.01091

**Published:** 2020-10-30

**Authors:** Wan-Yu Lo, Shou-Jie Wang, Huang-Joe Wang

**Affiliations:** ^1^Cardiovascular and Translational Medicine Laboratory, Department of Biotechnology, Hungkuang University, Taichung, Taiwan; ^2^Department of Life Sciences, National Chung Hsing University, Taichung, Taiwan; ^3^School of Medicine, China Medical University, Taichung, Taiwan; ^4^Division of Cardiovascular Medicine, Department of Internal Medicine, China Medical University Hospital, Taichung, Taiwan

**Keywords:** diabetes, high glucose, O-Linked *N*-acetylglucosamine transferase (OGT), protein *O*-GlcNAcylation, microRNA-146a-5p, endothelial inflammation

## Abstract

**Background and Aims:** Increased *O*-GlcNAc transferase (OGT)–induced O-linked *N*-acetylglucosamine (*O*-GlcNAc) post-translational modification is linked with diabetic complications. MicroRNA-146a-5p (miR-146a-5p) is a negative inflammatory regulator and is downregulated in diabetes. Here, we investigated the interaction between miR-146a-5p and OGT.

**Methods:** Human aortic endothelial cells (HAECs) were stimulated with high glucose (25 mM) and glucosamine (25 mM) for 24 h. Western blot, real time PCR, bioinformatics analysis, luciferase reporter assay, miR-146a-5p mimic/inhibitor transfection, siRNA OGT transfection, miR-200a/200b mimic transfection, and OGT pharmacological inhibition (ST045849) were performed. The aorta from miR-146a-5p mimic-treated db/db mice were examined by immunohistochemistry staining.

**Results:** HG and glucosamine upregulated OGT mRNA and protein expression, protein *O*-GlcNAcylation, and IL-6 mRNA and protein expression. Real time PCR analysis found that miR-146a-5p was decreased in HG- and glucosamine-stimulated HAECs. This suggested that OGT-induced protein O-GlcNAcylation as a mechanism to downregulate miR-146a-5p. Bioinformatic miR target analysis excluded miR-146a-5p as a post-transcriptional regulator of OGT. However, a luciferase reporter assay confirmed that miR-146a-5p mimic bound to 3′-UTR of human OGT mRNA, indicating that OGT is a non-canonical target of miR-146a-5p. Transfection with miR-146a-5p mimic and inhibitor confirmed that miR-146a-5p regulated OGT/protein O-GlcNAcylation/IL-6 expression levels. Furthermore, OGT siRNA transfection, miR-200a/miR-200b mimic transfection, and ST045849 increased HG-induced miR-146a-5p expression levels, indicating that HG-induced miR-146a-5p downregulation is partially mediated through OGT-mediated protein *O*-GlcNAcylation. In *vivo*, intravenous injections of miR-146a mimic decreased endothelial OGT and IL6 expression in db/db mice.

**Conclusion:** A non-canonical positive feedback interaction between miR-146a-5p and OGT is involved in a vicious cycle to aggravate HG-induced vascular complications.

## Introduction

Hyperglycemia is a major contributor to diabetic vascular disease. Hyperglycemia causes multiple tissue damages via mitochondria over-production of reactive oxygen species (ROS) and activation of hexosamine biosynthesis pathway (HBP) flux (Giacco and Brownlee, 2010). Glucose can divert different nutrients (including glucose, fatty acid, amino acid, and nucleotide) through HBP to regulate dynamic protein *O*-GlcNAcylation levels (Chen et al., 2019). Protein *O*-GlcNAcylation is an important post-translational modification to combat various cellular stresses (Issad et al., 2010). Two enzymes are known to control the protein *O*-GlcNAcylation expression levels. First, O-linked *N*-acetylglucosamine (*O*-GlcNAc) can be removed from *O*-GlcNAcylated proteins by β-*N*-acetylglucosaminidase (OGA) to decrease protein *O*-GlcNAcylation levels. Second, *O*-GlcNAc transferase (OGT) can add *O*-GlcNAc to the threonine and serine residues of targeted proteins to increase protein *O*-GlcNAcylation levels (Ma and Hart, 2014). Aberrant *O*-GlcNAcylation was closely linked with the pathophysiology of diabetes-related cardiomyopathy, retinopathy and nephropathy (Semba et al., 2014; Banerjee et al., 2016; Gellai et al., 2016; Ducheix et al., 2018). Previously, we also reported that protein *O*-GlcNAcylation upregulation was responsible for exacerbated endothelial inflammation in high glucose (HG) -stimulated human aortic endothelia cells (HAECs) (Lo et al., 2018).

MicroRNAs (miRs) play an essential role in regulating the post-transcriptional response in diabetes (Shantikumar et al., 2012; Guay and Regazzi, 2013). Among the multiple miRs, miR-146a-5p was identified as an anti-inflammatory regulator targeting signal molecules of nuclear factor kappa-light-chain enhancer of activated B cells (NF-κB) pathway (Taganov et al., 2006). As an inflammatory brake, stimulations with lipopolysaccharide, tumor necrosis factor-α, interleukin-8, and interleukin-1β in different cells can increase miR-146a-5p expression to inhibit the intense inflammation induced by various stimulants (Taganov et al., 2006; Li et al., 2012; Cheng et al., 2013). However, decreased production of miR-146a-5p was reported in diabetes-associated studies. Feng et al. (2011) reported that HG downregulated miR-146a expression in the human umbilical vein endothelial cells, and both streptozotocin-induced type 1 diabetic rats and db/db type 2 diabetic mice showed decreased miR-146a expression in the retina, heart, and kidney tissues. Xu et al. (2012) reported that wounds of diabetic mice expressed significantly downregulated miR-146a levels. Emadi et al. (2014) reported that miR-146a expression was downregulated in the aorta of streptozotocin-induced diabetic rats. Wang L. et al. (2014) reported that HG downregulated miR-146a expression in cultured dorsal root ganglions. We also reported that HG downregulated miR-146a expression in HAECs (Wang H.J. et al., 2014; Lo et al., 2017). These evidences highlight the importance of downregulated miR-146a in the pathogenesis of diabetes. In addition, human samples also showed miR-146a downregulation in the plasma (Assmann et al., 2018) and peripheral blood molecular cells (Yang et al., 2015) of type 1 diabetic patients. A recent meta-analysis concluded that miR-146a expression was downregulated in the whole blood and peripheral blood mononuclear cells of type 2 diabetic patients (Alipoor et al., 2017).

Although endothelial inflammation in HG-stimulated HAECs is closely linked to the miR-146a-5p downregulation and protein *O*-GlcNAcylation upregulation, the interaction between them is not yet clear. In this study, we investigated the interaction between miR-146a-5p and OGT in HG-stimulated (HAECs) and conducted an *in vivo* study to investigate the aortic endothelial tissues in miR-146a-5p mimic-treated db/db diabetic mice.

## Materials and Methods

### Endothelial Cell Culture

Human aortic endothelial cells were purchased from Cell Applications, Inc. (San Diego, CA, United States), and cultivated as previously described (Wang H.J. et al., 2014). High glucose (25 mM) and glucosamine (25 mM) stimulation for 24 h to HAECs were performed in various experiments. Mannitol (25 mM) was used as the osmotic control.

### Real-Time Polymerase Chain Reaction (PCR)

The total mRNA was extracted by PureLink^TM^ RNA Mini Kit (Thermo Fisher Scientific) and the expression levels in HAECs were analyzed by real-time PCR. The first-strand cDNA was synthesized by SuperScript^®^ III First-Strand Synthesis SuperMix (Invitrogen, Carlsbad, CA, United States). All PCR reactions were performed by StepOnePlus Real-Time PCR instrument (Applied Biosystems, Foster, CA, United States), as previously described (Pai et al., 2017; Wang and Lo, 2017). The beta-actin was selected as the internal control. The primer sequences for OGT, interleukin-6 (IL-6), TNF-α, OGA, and beta-actin were listed as below.

OGT Forward primer: 5′-GCAGCAGGACCAATTAC CTC-3′
Reverse primer: 5′-GCATACGTTTCGTTGGTTCTG-3′
IL-6 Forward primer: 5′-GATGAGTACAAAAGTCCT GATCCA-3′
Reverse primer: 5′-CTGCAGCCACTGGTTCTGT-3′
TNF-α Forward primer: 5′-CCTTTCTGCGAGAGGG AAC-3′
Reverse primer: 5′-CACCTTGCAGGAGTTGTCAGT-3′
OGA Forward primer: 5′-TGTGGTGGAAGGATTTT ATGG-3′
Reverse primer: 5′-TCATCTTTTGGGGCATACAAG-3′
Beta-actin Forward primer: 5′-ACCATGTACCCTGGCA TTG-3′
Reverse primer: 5′-AGGAAAGACACCCACCTTGA-3′

### Western Blot

Each protein sample was separated by sodium dodecyl sulfate polyacrylamide gel electrophoresis and transferred onto a Immobilon-P Transfer Membrane (Millipore, United States) using a Semi-Dry and Rapid Blotting System (Bio-Rad), as previously described (Lo et al., 2018). Protein expression levels were normalized to beta-actin expression. Primary antibodies against beta-actin (1:5000, Santa Cruz Biotechnology, Santa Cruz, CA, United States), OGT (1:1000; Abcam, Cambridge, MA, United States), *O*-GlcNAcylation (RL2, 1:1000; Abcam), and IL-6 (1:1000, Cell Signaling Technology, Danvers, MA, United States) were used. Immunostaining was performed using SuperSignal West Pico Chemiluminescent Substrate (Thermo Fisher, Rockford, IL, United States) for OGT, and *O*-GlcNAcylation (RL2) and SuperSignal West Femto Maximum Sensitivity Substrate (Thermo Fisher) for IL-6 (Lo et al., 2018).

### TaqMan miR Assay for miR-146a-5p

Total RNA, including miRs, were extracted from HAECs using High Pure miRNA Isolation Kit (Invitrogen, Carlsbad, CA, United States). The variable miRs and RNU6B-specific cDNA were synthesized according to the TaqMan microRNA assay kit (RNU6B: Cat No. 602003; miR-146a-5p: Cat No. P02594635) and protocol (Applied Biosystems). RNU6B was used as the internal control. Quantitative real-time PCR was performed using the StepOnePlus Real-Time PCR instrument (Applied Biosystems).

### Transfection of miR-146a-5p Mimic, miR-146a-5p Inhibitor, and miR-200a/200b Mimics

miR-146a-5p mimic, miR-146a-5p inhibitor, miR-200a mimic, miR-200b mimic and negative control (NC) were transfected into HAECs, as previously described (Lo et al., 2017, 2018). After miR-146a-mimic or inhibitor transfection, HAECs were treated with HG for 24 h, after which the expression levels of OGT mRNA, OGT, protein *O*-GlcNAcylation, IL-6 gene and IL-6 protein were analyzed. After miR-200a/200b mimics transfection, HAECs were treated with HG for 24 h, after which the expression levels of miR-146a-5p were analyzed.

### Luciferase Reporter Assay

A segment of human OGT 3′-UTR that includes miR-146a-5p binding site was constructed into the pmirGLO vector (Promega, Madison, WI, United States) and was named as pmirGLO OGT-3′-UTR. As a mutant control, another segment of human OGT-3′-UTR that includes the mutant seed sequence binding site of miR-146a-5p was constructed into the pmiGLO vector and was named as pmirGLO OGT-mutant-3′-UTR. HAECs were cotransfected with 1 μg of constructed plasmids and 100 nM of miR-146a-5p mimic or NC using Lipofectamine^TM^ 2000 (Invitrogen, Carlsbad, CA, United States). After 24 h of transfection, cells were harvested to determine luciferase activity using the Luciferase Assay System Kit (Promega, E1500), as previously described (Lo et al., 2018).

### OGT Gene Silencing

For OGT gene knockdown, HAECs were transfected with 100 nM human OGT siRNA (GeneDirex, Keelung, Taiwan), and the efficiency of siRNA OGT was previously described (Lo et al., 2018). Scrambled NC siRNA was included as a negative control. After OGT siRNA transfection, the medium was changed to the fresh endothelial cell growth medium, and HAECs were stimulated with HG for 24 h. After HG treatment, expression levels of miR-146a-5p were determined.

### Inhibition of OGT

For pharmacological inhibition of OGT, HAECs were pretreated with 20 mM ST045849 (R&D Systems, Minneapolis, MN, United States) overnight. DMSO was included as a vehicle control. After OGT inhibition, the medium was changed to the fresh endothelial cell growth medium, and HAECs were stimulated with HG for 12 h. After HG treatment, expression levels of miR-146a-5p were determined.

### Type 2 db/db Diabetic Mouse Model Experiments

Animal studies were approved by the Hungkuang University Institutional Animal Care and Use Committee. Male db/db mice were obtained from the National Laboratory Animal Center (Nangang, Taipei, Taiwan). Fourteen-week-old db/db mice were injected with 100 μl miR-146a-5p mimic or negative control (13 μg per week, three times) by tail-vein injection method, using equal volume mixtures of Lipofectamine^TM^ 2000 and miR-146a-5p mimic or negative control. The Lipofecamine^TM^2000 control db/db group received equal volume mixtures of Lipofecamine^TM^2000 and PBS. Mice were sacrificed by CO2 narcosis after 3 weeks. Aortic tissue was cautiously excised and fixed with formalin solution. Aorta paraffin sections were prepared for immunohistochemistry (IHC) staining.

### IHC Staining

Briefly, after deparaffinization and rehydration, the aortic sections were rinsed by PBS and then preheated with the antigen retrieval buffer (100 mM Tris, 5% (w/v) urea, adjust pH to 9.5 with HCl) to 95°C for 10 min. After cooling and washing with 1X PBS, cells were permeabilized with 0.1% Triton X-100 in PBS at RT for 10 min. Next, the sections were blocked and incubated with anti-OGT (ab96718; Abcam; dilution 1:100) or anti-IL-6 (Santa Cruz; dilution 1:100) at 4°C overnight before being processed with the commercial kit (VECTASTAIN^®^ Elite^®^ ABC Kit, Vector Laboratories, CA, United States). Subsequently, the sections were counterstained with hematoxylin (Abcam, ab22035) for histological evaluation. The sections were covered with a coverslip using mounting solution (VECTASHIELD^®^Vibrance^TM^). Finally, these immunosignals were examined under a microscope (Nikon ECLIPSE E200, Tokyo) using the Image J software.

### Statistical Analysis

Statistical analyses were performed using SPSS 12.0 statistical software (SPSS Inc., Chicago, IL, United States). Data are presented as the mean ± SEM. Pair-wise comparisons were performed by a Student’s *t*-test. Three or more groups were compared by one-way analysis of variance with post-hoc Tukey tests. Significant differences were defined as *p* < 0.05.

## Results

### HG and Glucosamine Increase OGT/Protein *O*-GlcNAcylation/IL-6 Expression and Decrease miR-146a-5p Expression

**FIGURE 1:**
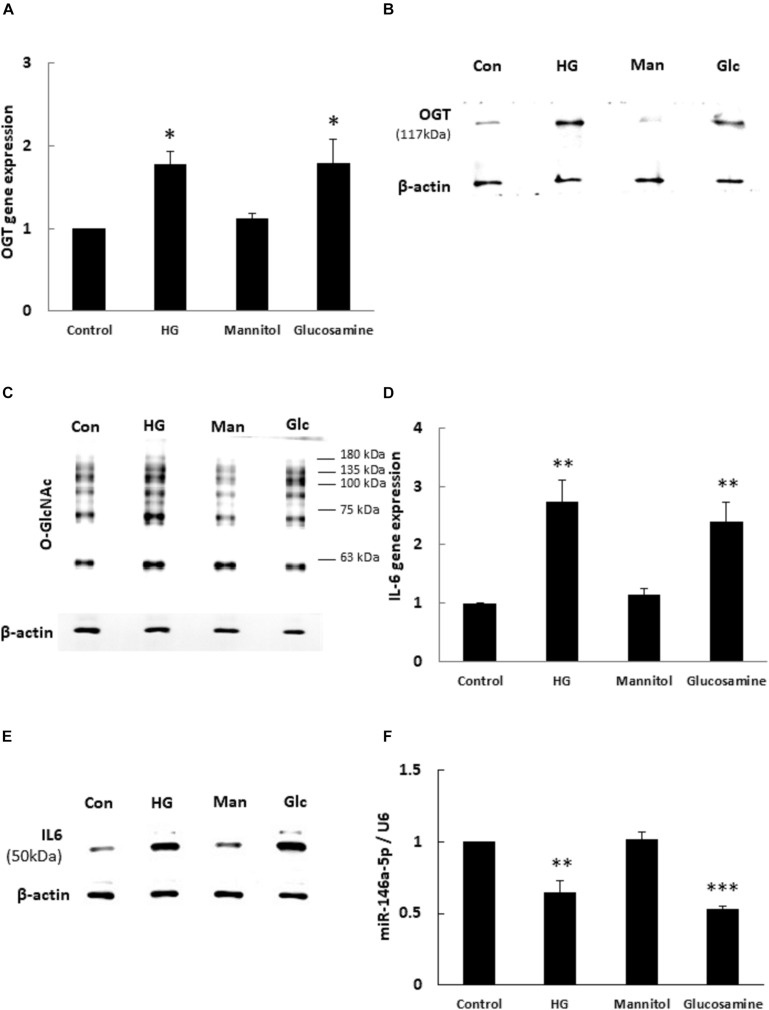
**(A–F)** High glucose (HG) and glucosamine (Glc) increased OGT/protein *O*-GlcNAcylation/IL-6 expression and decreased miR-146a expression. **(A)** HAECs were stimulated with HG (25 mM) and glucosamine (25 mM) for 24 h. Real-time PCR revealed that HG and glucosamine stimulation induced 1.77- and 1.79-fold increases, respectively, in OGT mRNA levels. Mannitol (25 mM) did not modulate OGT mRNA levels. *N* = 5. *p < 0.05 compared to control. **(B,C)** Stimulation of HAECs with HG and glucosamine for 24 h induced a significant increase in OGT protein and protein *O*-GlcNAcylation (as detected by RL2 antibody) expression. The blot is representative of three independent experiments. **(D)** Stimulation of HAECs with HG and glucosamine for 24 h induced 2.73- and 2.39-fold increases in IL-6 mRNA levels. *N* = 5. **p < 0.01 compared to control. **(E)** Stimulation of HAECs with HG and glucosamine for 24 h induced a significant increase in IL-6 expression. The blot is representative of three independent experiments. **(F)** Real-time PCR showed that HG and glucosamine stimulation for 24 h decreased miR-146a-5p expression levels to 65 and 53% of the control level, respectively. Mannitol did not modulate miR-146a-5p expression levels. miR expression levels were normalized to U6 expression. *N* = 3. **p < 0.01 compared with control. ***p < 0.001 compared with control.

To determine whether OGT and protein *O*-GlcNAcylation expression levels were changed with HG and glucosamine, we first examined the effects of HG and glucosamine stimulation on endothelial OGT and protein *O*-GlcNAcylation expression. After 24 h treatment, HG and glucosamine stimulation caused significant (1.77- and 1.79-fold, respectively) increases in OGT mRNA expression in HEACs as compared with the unstimulated control (Figure 1A). In contrast, the osmotic control mannitol did not modulate the mRNA expression levels of OGT. HG and glucosamine stimulation for 24 h also significantly increased OGT protein and protein *O*-GlcNAcylation expression levels (Figures 1B,C). IL-6 is a pro-inflammatory cytokine that contributes to atherosclerotic disease development (Schuett et al., 2009). HG and glucosamine stimulation for 24 h caused 2.73- and 2.39-fold increases in IL-6 mRNA expression, respectively (Figure 1D).

Similarly, HG and glucosamine stimulation for 24 h caused 1.3- and 1.3-fold increases in TNF-α mRNA expression, respectively (Supplementary Figure 1A, *^∗∗∗^P* < 0.001). The expression of IL-6 protein was significantly upregulated after 24 h HG and glucosamine (Figure 1E). In order to examine whether miR-146a-5p expression levels were associated with altered OGT and protein *O*-GlcNAcylation expression, the HAECs were treated with HG and glucosamine for 24 h. As shown in Figure 1F, real-time PCR analyses showed that HG and glucosamine stimulation for 24 h decreased miR-146a-5p expression levels to 65 and 53% of the control level, respectively. Mannitol did not change miR-146a-5p expression levels. These data identified a negative correlation between OGT/protein *O*-GlcNAcylation/IL-6 expression levels and miR-146a-5p expression levels.

### Human OGT Is a Non-canonical Target of miR-146a-5p

**FIGURE 2:**
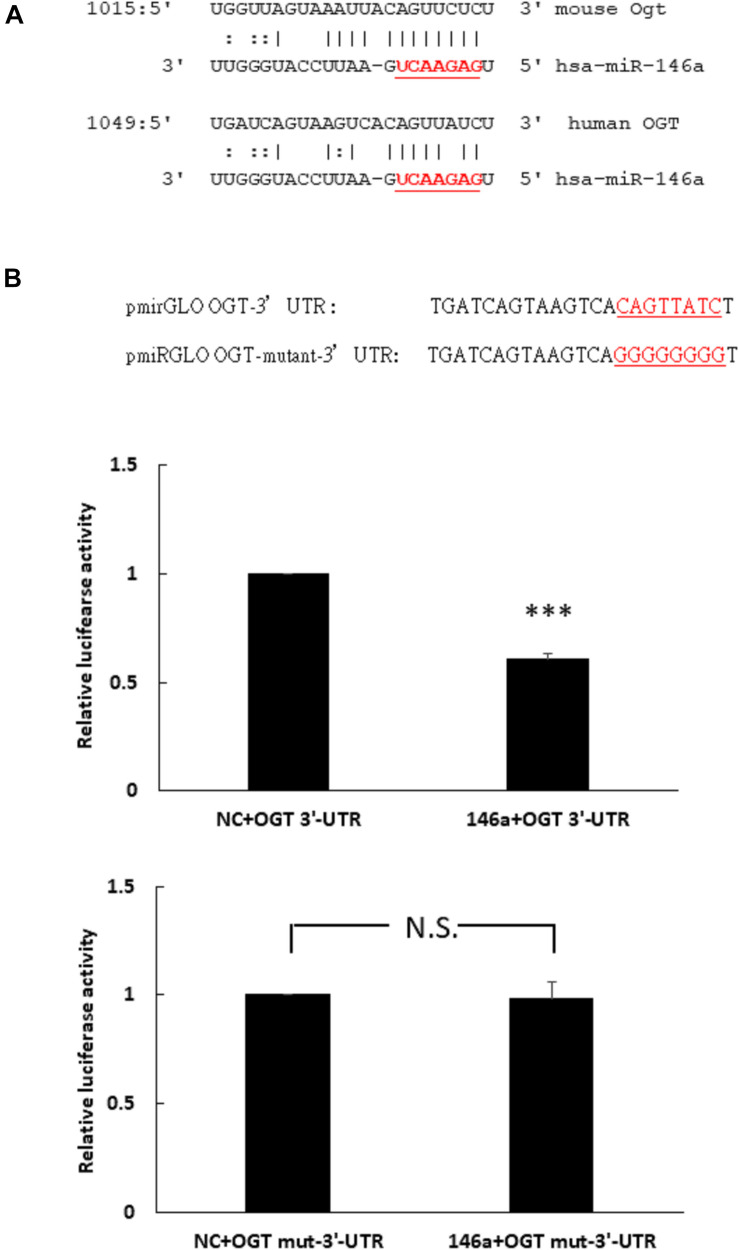
**(A,B)** Human OGT is a non-canonical target of miR-146a-5p. **(A)** Bioinformatic miR target analysis identified partial seed sequence match between miR-146a-5p and the 3′-UTR of human OGT mRNA. However, mouse Ogt showed perfect seed sequence homologies between miR-146a-5p and the 3′-UTR of mouse OGT mRNA. **(B)** A luciferase reporter assay showed that co-transfection of the miR-146a-5p mimic and pmirGLO-OGT-3′-UTR could downregulate the relative luciferase activity to 61% of the control luciferase signal, *N* = 5, ^∗∗∗^*p* < 0.001 compared with the negative control. Co-transfection of the miR-146a-5p mimic and pmirGLO-OGT-mutant-3′-UTR did not alter the relative luciferase activity. *N* = 5, N.S., not significant, compared to the negative control (NC).

To determine whether miR-146a-5p was a potential binding partner of the 3′- untranslated region (3′-UTR) of human OGT mRNA, *in silico* analyses using miRDB and miRanda-mirSVR database were performed. Neither database identified miR-146a-5p as a potential binding partner of the 3′-UTR of human OGT. However, careful manual sequence alignment identified a partial seed sequence match between miR-146a-5p and the 3′-UTR of human OGT mRNA. Interestingly, a perfect seed sequence match between miR-146a-5p and the 3′-UTR of mouse OGT mRNA were found (Figure 2A). To investigate whether miR-146a-5p could interact with the 3′-UTR of human OGT mRNA in a non-canonical way, a luciferase reporter assay was performed. As shown in Figure 2B, co-transfection of pmirGLO-OGT-3′-UTR and the miR-146a-5p mimic resulted in a decrease in the relative luciferase activity to 61% of that in the negative control, confirming the direct binding of miR-146-5p to the 3′-UTR of human OGT mRNA in a non-canonical way. In contrast, co-transfection of the miR-146a-5p mimic and pmirGLO-OGT-mutant-3′-UTR did not alter the relative luciferase activity.

### miR-146a-5p Mimic and Inhibitor Regulate HG-Induced OGT, Protein *O*-GlcNAcylation, and IL-6 Expression

**FIGURE 3:**
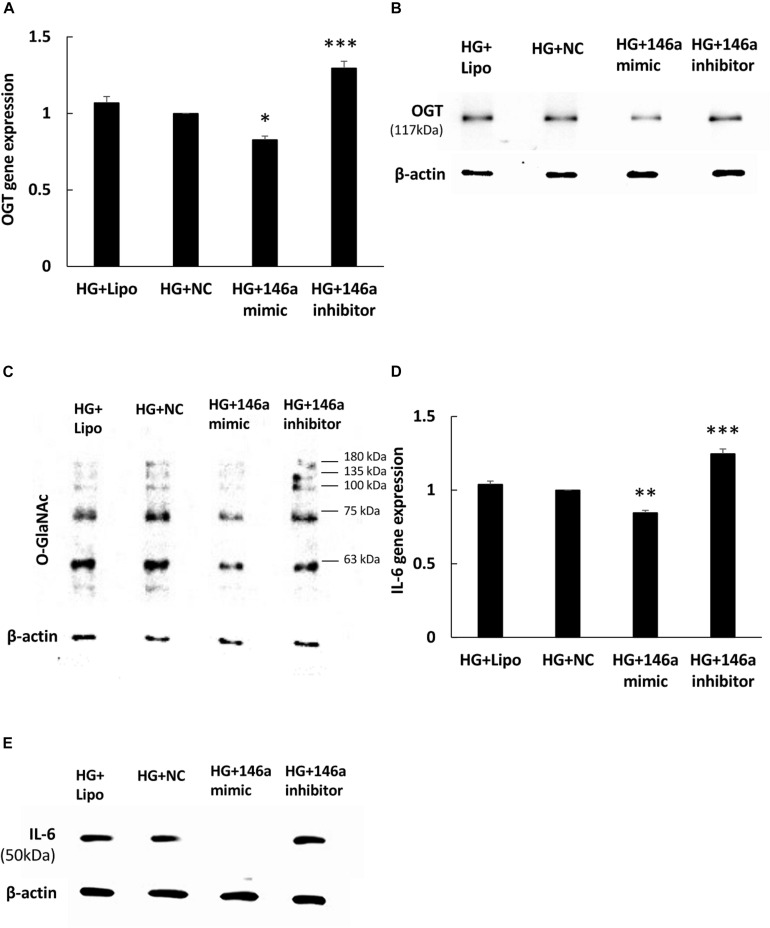
**(A–E)** miR-146a-5p mimic and inhibitor regulated HG-induced OGT mRNA and protein expression, protein *O*-GlcNAcylation, and IL-6 mRNA and protein expression. **(A)** Transfection of miR-146a-5p mimic inhibited HG-induced OGT mRNA expression to 83% of the control level. *N* = 4. ^∗^*p* < 0.05 compared with the HG-treated HAECs transfected with a negative control (HG + NC). Conversely, transfection of miR-146a-5p inhibitor enhanced HG-induced OGT mRNA expression by 1.30-fold. *N* = 4. ^∗∗∗^*p* < 0.001 compared with HG + NC. **(B,C)** The stimulatory effect of HG on OGT protein and protein *O*-GlcNAcylation expression levels were significantly inhibited in the miR-146a-5p mimic-transfected HAECs compared to the HAECs transfected with negative control. Conversely, transfection of miR-146a-5p inhibitor increased HG-induced OGT protein and protein *O*-GlcNAcylation expression levels. The blot is representative of three independent experiments. **(D)** Transfection of miR-146a-5p mimics inhibited HG-induced IL-6 mRNA expression to 85% of the control level, respectively. *N* = 4. ^∗∗^*p* < 0.01 compared with HG + NC. Conversely, transfection of miR-146a-5p inhibitor increased HG-induced OGT mRNA expression by 1.25-fold. *N* = 4. ^∗∗∗^*p* < 0.001 compared with HG + NC. **(E)** The stimulatory effect of HG on IL-6 protein expression levels were significantly inhibited in the miR-146a-5p mimic-transfected HAECs than in the HAECs transfected with negative control. Conversely, transfection of miR-146a-5p inhibitor enhanced HG-induced IL-6 protein expression levels. The blot is representative of three independent experiments.

To investigate whether miR-146a-5p could regulate OGT mRNA expression, transfection assays with 146a-5p mimic and inhibitor were performed. As shown in Figure 3A, transfection of miR-146a-5p mimic in HG-stimulated HAECs decreased OGT mRNA expression level to 83% of that in the negative control. By contrast, transfection of miR-146a-5p inhibitors in HG-stimulated HAECs increased OGT mRNA expression level to 1.30-fold of that in the negative control. OGA is another important regulator to control protein *O*-GlcNAcylation expression levels. *In silico*, OGA is not predicted as a target of miR-146a-5p. Our data also showed that OGA was not sensitive to the miR-146a-5p mimic (Supplementary Figure 1B, N.S. not significant). As compared with the negative control group, transfection of the miR-146a-5p mimic downregulated OGT protein expression and was associated with reduced protein *O*-GlcNAcylation in HG-stimulated HAECs (Figures 3B,C). In contrast, transfection of the miR-146a-5p inhibitor upregulated OGT protein expression and was associated with enhanced protein *O*-GlcNAcylation, as compared with the negative control group (Figures 3B,C). To determine whether altered OGT and protein *O*-GlcNAcylation expression were associated with altered endothelial inflammation in miR-146a-5p mimic and inhibitor-transfected HG-stimulated HAECs, we measured the IL-6 gene expression level. As showed in Figure 3D, transfection of miR-146a-5p mimic inhibited HG-induced IL-6 mRNA expression to 85% of negative control expression levels. In contrast, transfection of miR-146a-5p inhibitor increased HG-induced IL-6 mRNA expression to 1.25-fold of negative control expression levels (Figure 3D). *In silico*, TNF-α is not predicted as a target of miR-146a-5p. However, transfection of miR-146a-5p mimic inhibited HG-induced TNF-α expression to 68% of negative control expression levels, suggesting that an indirect regulatory role of miR-146-5p existed in HG-stimulated HAECs as a negative regulator of NF-κB pathway (Supplementary Figure 1C, *^∗∗∗^P* < 0.001). IL-6 protein expression was also decreased by transfection of the miR-146a-5p mimic and increased by transfection of miR-146a-5p inhibitor in HG-stimulated HAECs (Figure 3E). These results indicate that miR-146a-5p can regulate protein *O*-GlcNAcylation levels via a post-transcriptional mechanism to control OGT expression levels.

### HG-Induced miR-146a-5p Downregulation Is Inhibited by OGT siRNA Transfection, miR-200a/200b Mimic Transfection, and ST045849

**FIGURE 4:**
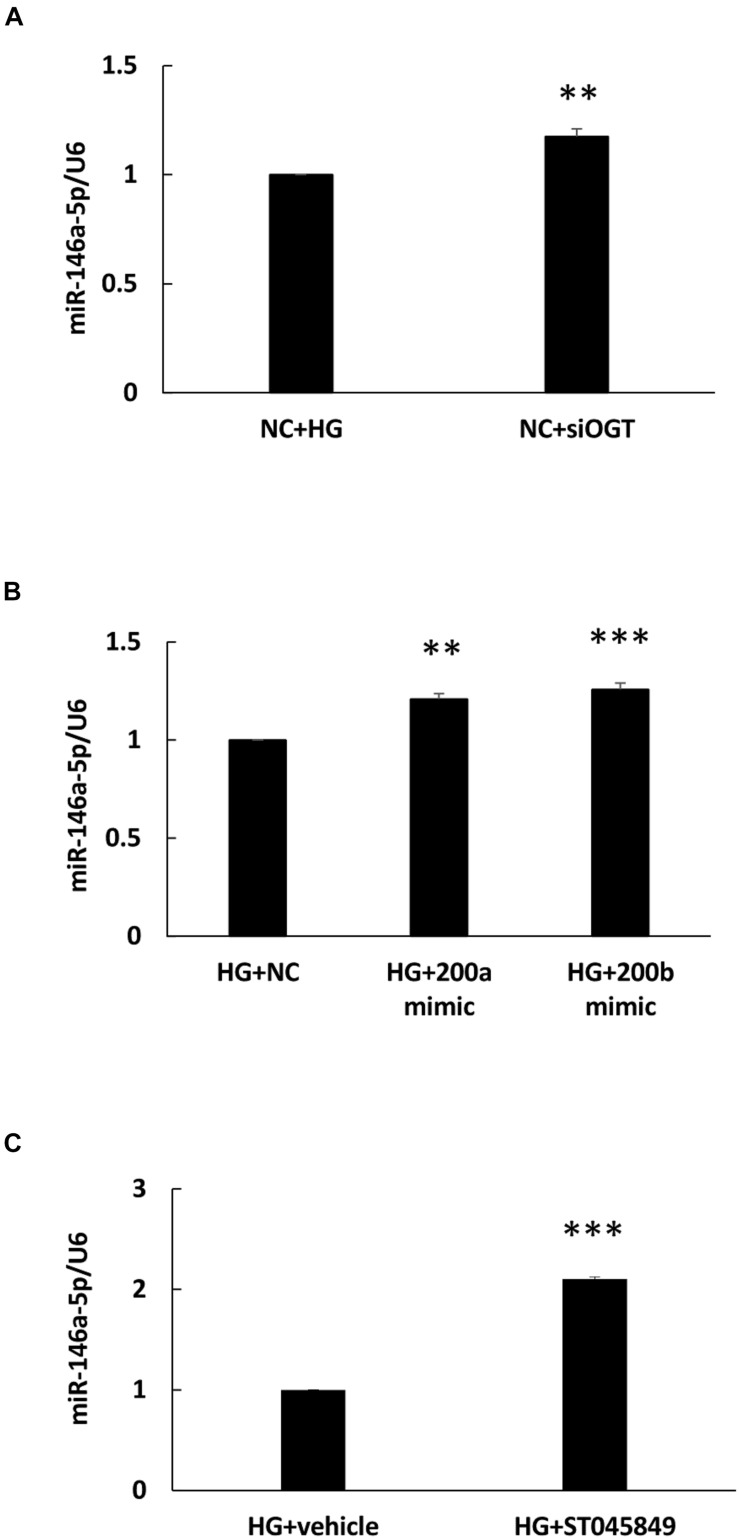
**(A–C)** HG-treated endothelial miR-146a expression level were increased by OGT siRNA transfection, miR-200a/miR-200b mimic transfection, and ST045949. **(A)** Real-time PCR revealed that OGT siRNA transfection induced 1.18-fold increases in miR-146a-5p expression levels, *N* = 3. **p < 0.01 compared with HG-treated HAECs transfected with a negative control (HG + NC). **(B)** Real-time PCR revealed that miR-200a/200b mimics transfection induced 1.21- and 1.26-fold increases in miR-146a-5p expression levels. *N* = 4. **p < 0.01 compared with HG + NC. ***p < 0.001 compared with HG + NC. **(C)** Real-time PCR revealed that ST045849 pretreatment induced 2.1-fold increases in miR-146a-5p expression levels. *N* = 5. ***p < 0.001 compared with HG-treated HAECs pretreated with vehicle.

To determine whether miR-146a-5p expression was modulated with HG-induced protein *O*-GlcNAcylation, an OGT-specific siRNA transfection experiment was done. As shown in Figure 4A, OGT siRNA transfection significantly caused a 1.18-fold increase in miR-146a-5p expression levels in HG-stimulated HAECs, as compared with HG-stimulated HAECs transfected with the scrambled negative control. Previously, ROS-sensitive miR-200a/200b were identified as seed sequence match, canonical post-transcriptional regulators of OGT (Lo et al., 2018). In this study, transfection with miR-200a and 200b mimics also significantly caused a 1.21- and 1.26-fold increase in miR-146a-5p expression levels, respectively, in HG-stimulated HAECs, as compared with HG-stimulated HAECs transfected with the negative control (Figure 4B). For pharmacological inhibition of OGT, pretreatment with ST045849 significantly caused a 2.1-fold increase in miR-146a-5p expression levels in HG-stimulated HAECs, as compared with HG-stimulated, vehicle-pretreated HAECs (Figure 4C). These data indicated that HG-induced miR-146a-5p downregulation was partially mediated through OGT-controlled protein *O*-GlcNAcylation.

### The miR-146a-5p Mimic Decreases Endothelial OGT and IL-6 Expression in db/db Diabetic Mice

**FIGURE 5:**
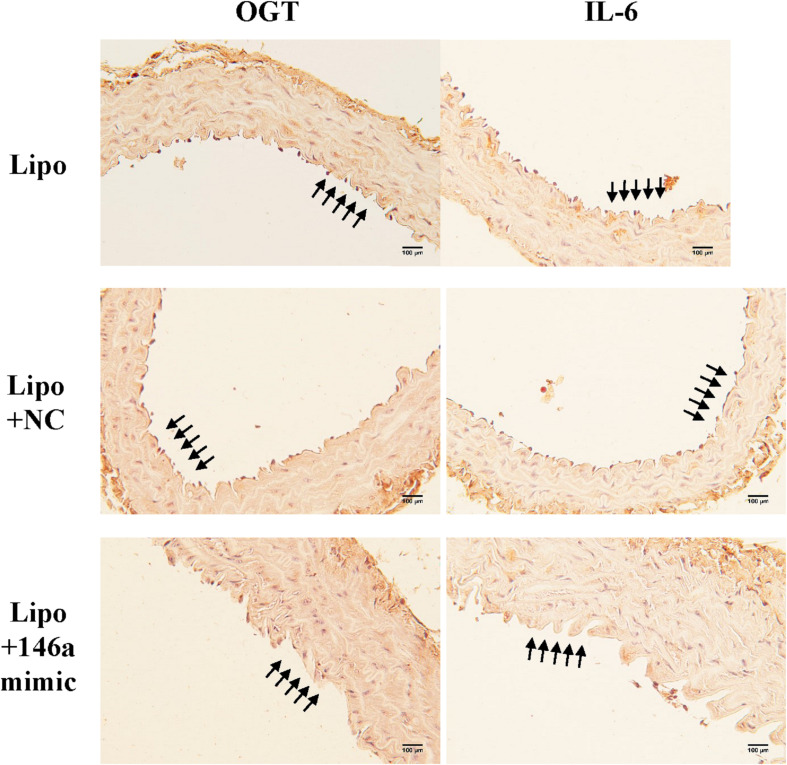
The miR-146a-5p mimic decreased endothelial OGT and IL-6 expression in type 2 db/db diabetic mice. Immunohistochemistry staining of OGT and IL-6 in thoracic aorta tissue. Representative images showed that the immunoreactivities of endothelial OGT and IL-6 in the aortic endothelial layers were decreased in the miR-146a-5p mimic-treated db/db mice, as compared to negative control (NC)-treated db/db or Lipo-treated db/db mice. *N* = 3 per group. Scale bar = 100 μm. Lipo, Lipofectamine^TM^2000.

**FIGURE 6:**
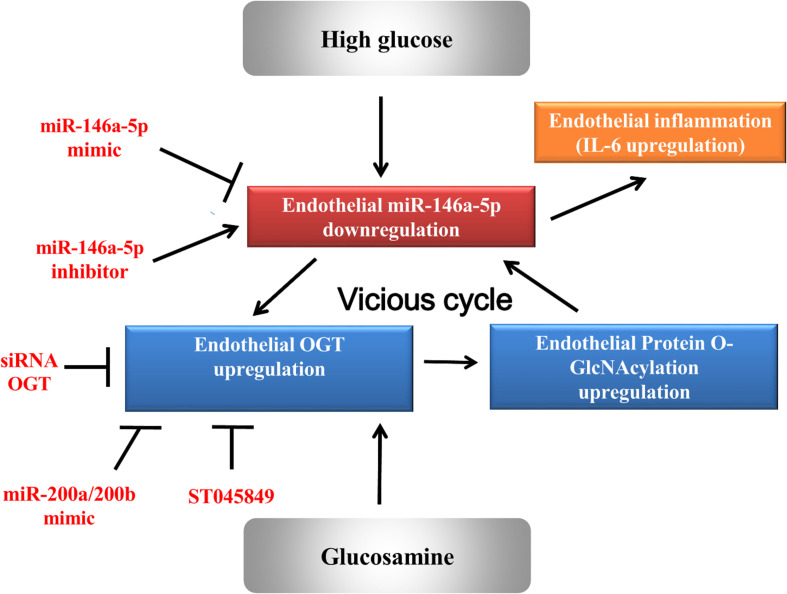
A proposed vicious cycle interaction between endothelial miR-146a-5p and human OGT.

In order to investigate the effect of the miR-146a-5p mimic on the expression of endothelial OGT and IL-6 in db/db mice, we performed IHC staining on aortic tissues. As shown in Figure 5, the immunoreactivities of OGT and IL-6 in the aortic endothelial layers were significantly downregulated in the miR-146a-5p mimic-treated db/db mice, as compared to the negative control or vehicle control-treated db/db mice. This suggests that miR-146a-5p mimics possess a potential value in treating diabetic vascular disease through the downregulation of OGT and IL-6. In diabetes with chronic hyperglycemia, the proposed interaction between endothelial miR-146a-5p and OGT/protein *O*-GlcNAcylation is illustrated in Figure 6.

## Discussion

The study demonstrates that HG and glucosamine can upregulate OGT expression, protein *O*-GlcNAcylation levels, and IL-6 in HAECs by downregulating miR-146a-5p expression. Among the multiple mechanisms responsible for diabetic-induced chronic inflammation, a decrease in miR-146a-5p is a consistent finding that explains a persistent pro-inflammatory status in diabetes. We found that a decrease in miR-146a-5p in HG-stimulated HAECs can upregulate the OGT and protein *O*-GlcNAcylation expression levels via a non-canonical post-transcriptional regulatory mechanism. The upregulated OGT and protein *O*-GlcNAcylation expression levels, in turn, sustain miR-146a-5p downregulation and cause miR-146a-5p downstream targets (i.e., OGT and IL-6) upregulation and endothelial inflammation. Both genetic and pharmacological inhibition of OGT confirmed that a mechanistic interaction exists between OGT and miR-146a-5p. This positive feedback loop (as illustrated in Figure 6) can aggravate endothelial inflammation in diabetic vascular disease. As a therapeutic potential drug, our *in vitro* and *in vivo* experiments indicated that miR-146a-5p can treat diabetic vascular disease.

Increased protein *O*-GlcNAcylation has been linked to diabetic pathophysiology (Lima et al., 2012; Bond and Hanover, 2013). Du et al. (2003) reported that HG-induced oxidative stress inhibited glyceraldehyde-3-phosphate dehydrogenase activity, resulting in activation of HBP and NF-κB pathway in bovine aortic endothelial cells. Donovan et al. (2014) reported that *O*-GlcNAcylation of Sp1 increased HG-induced VEGF-1 upregulation in human retinal pigment epithelial cells. Similarly, Zhang et al. (2017) reported that *O*-GlcNAcylation of Sp1 mediated HG-induced intercellular adhesion molecule 1 (ICAM-1) upregulation in human umbilical vein endothelial cells and rat capillary endothelial cells. In rat vascular smooth muscles, Yang et al. (2008) reported that *O*-GlcNAcylation of Threonin-352 of NF-κB p65 interfered with the interaction between NF-κB and Iκ-B, a condition that was followed by increased transcriptional activity of NF-κB. Interestingly, they found a longer half-life of *O*-GlcNAcylated NF-κB in the nucleus, compared with unmodified NF-κB and considered it as one of mechanisms to sustain NF-κB activation in diabetes. In rat mesangial cells, Park et al. (2014) reported that HG-induced *O*-GlcNAcylated carbohydrate response element-binding protein stimulated lipogenesis and fibrosis, suggesting that a role of *O*-GlcNAcylation in the development of diabetic nephropathy. In human tissue, Federici et al. (2002) observed that carotid atherosclerotic plaques from type 2 diabetic patients had more protein *O*-GlcNAcylation than non-diabetic patients. They reported that HG and glucosamine not only increased protein *O*-GlcNAcylation and but also impaired insulin signaling in human coronary endothelial cells (Federici et al., 2002). Recently, we also reported that HG-induced ICAM-1, vascular cell adhesion molecule 1, and *E*-selectin expression were positively correlated with protein *O*-GlcNAcylation expression levels in HG-stimulated HAECs (Lo et al., 2018). These multiple evidences suggested that *O*-GlcNAcylation as a detrimental modification to sustain the diabetic complications.

Glucose is metabolized to fructose-6-phosphate, and it is then fluxed into HBP by converting fructose-6-pohosphate into gucosamine-6-phosphate using a rate-liming glutamine:fructose 6-phosphate amidotransferase (GFAT) enzyme (Bond and Hanover, 2015). Experimentally, protein *O*-GlcNAcylation expression levels can be increased by high concentrations of glucose or glucosamine to bypass the GFAT (Federici et al., 2002; James et al., 2002; Allison et al., 2012). In this study, we demonstrated that HG and glucosamine not only increased protein *O*-GlcNAcylation via the induced OGT expression, but also downregulated an essential negative NF-κB regulator: the miR-146a-5p. To our knowledge, this inverse relationship between upregulated *O*-GlcNAcylation and downregulated miR-146a-5p is a novel finding in the literature. Furthermore, a mechanistic insight pointed that OGT/protein *O*-GlcNAcylation expression levels played a key role in regulating endothelial miR-146a-5p expression level, as HG-induced miR-146a-5p downregulation was reversed after OGT depletion by siRNA, OGT depletion by the canonical OGT regulators (i.e., miR-200a/200b mimics) (Lo et al., 2018), and OGT pharmacological inhibition by ST045849.

We selected two different web-based miRDB and miRanda-mirSVR mRNA target predication tools. Both predication tools failed to identify human OGT as a potential miR-146a-5p target. Common features of these predication tools depend on seed match, evolutionary conservation of a sequence across species, miRNA site accessibility to the mRNA target, and favorable miRNA-target mRNA thermodynamics (Peterson et al., 2014). The seed sequence is defined as first 2–8 nucleotides starting at the 5′ end of microRNA and most predication tools required a Watson-Crick match [i.e., adenosine (A)-uracil (U) and guanine (G)-cytosine (C)] between seed sequence of microRNA and its target mRNA (Lewis et al., 2005; Peterson et al., 2014). A single G:U wobble in the seed sequence was detrimental in the miRNA regulation but was allowed (Doench and Sharp, 2004). Brennecke et al. (2005) reported that a single 8-mer seed (miRNA position 1–8) was sufficient to give strong regulation by the miRNA and a single 7-mer seed (miRNA position 2–8) was also functional, albeit less efficient. Furthermore, the miRNA regulation was not effected by any mismatch between position 1, 9, 10, but strongly reduced in target regulation by any mismatch between position 2–8 (Brennecke et al., 2005). Therefore, current miRNA predication tools weight heavily on the seed sequence match rule (Saito and Saetrom, 2010; Peterson et al., 2014). Interesting, based on the seed sequence match, the 3′-UTR of mouse Ogt mRNA was identified as a potential binding partner of miR-146a-5p in the miRanda-mirSVR tool, but not in the miRDB tool. This finding made us perform careful manual sequence alignment, and we identified only partial seed sequence match between miR-146a-5p and the 3′-UTR of human OGT mRNA. Positions 2–8 of the seed sequence of miR-146-5p (5′-GAGAACU-3′) were not predicated to bind to the 3′-UTR of human OGT mRNA (3′-CUAUUGA-5′). However, positions 2–8 of the seed sequence of miR-146-5p (5′-GAGAACU-3′) were perfectly bound to the 3′-UTR of mouse Ogt mRNA (3′-CUCUUGA-5′) (Figure 2A). The nucleotide change in the seed sequence binding site (C -> A) from mouse Ogt mRNA to human OGT mRNA meant the current predication tools failed to pick up miR-146a-5p as a potential post-transcriptional regulator of human OGT mRNA. In this study, despite the lack of prediction of binding between human OGT and miR-146a-5p, the luciferase reporter assay and miR-146a-5p mimic/inhibitor transfection experiments confirmed a non-canonical regulation did exist between human OGT and miR-146a-5p.

Bioinformatic miR target predication tools have high false positive and false negative rates due to a tradeoff between sensitivity and specificity in different target prediction algorithms (Maziere and Enright, 2007). Although false positive results can be excluded through the subsequent laboratory works, the false negative results are less appreciated. In this study, predication tools weighted heavily on the seed sequence match rule clearly excluded the identification of human OGT as a target gene for the miR-146a-5p. The false negative results that are based on the seed sequence match rule were not infrequent and were documented in several recent works. Martin et al. (2014) reported that imperfect centered miRNA binding sites could mediate translational repression in the absence of seed match. Chi et al. (2012) reported that miR-124 in the brain was bound and regulated by G-bulge sites at position 5–6. They proposed a model that suggested position 6 as the pivot nucleotide to exert a transitional ‘nucleation bulge’ and subsequent functional bulge mRNA-miRNA interactions (Chi et al., 2012). Helwak et al. (2013) found more than 18,000 high confidence miRNA-mRNA interactions in a high-throughput mapping dataset. Although they found that most miRNA binding depended on the 5′-seed region, around 60% of seed interactions were non-canonical by the bulged or mismatched nucleotides in the seed sequence. In addition, 18% of miRNA-mRNA interactions were miRNA 3′-end dependent, with little evidence of 5′ seed match and some such non-canonical interactions were functional validated (Helwak et al., 2013). These studies pointed out that non-canonical miRNA–mRNA interactions are not infrequently and more prevalent than previously considered. Most current mRNA target predication tools considered only stringent-seed rules and inevitable missed many potential biologically important targets (Saito and Saetrom, 2010). In this study, a hint derived from the interaction between mouse Ogt mRNA and miR-146a-5p led us to identify a vicious cycle via the human OGT and miR-146a-5p interaction.

## Conclusion

This study confirmed that the miR-146a-5p regulation is closely linked to the OGT/protein *O*-GlcNAcylation regulation in a vicious cycle loop to aggravate endothelial inflammation in HG-stimulated HAECs, and both *in vitro* and *in vivo* experiments highlight the therapeutic potential of miR-146a-5p in treating diabetic vascular disease.

## Data Availability Statement

All datasets generated for this study are included in the article/Supplementary Material.

## Ethics Statement

The animal study was reviewed and approved by the Hungkuang University Institutional Animal Care and Use Committee.

## Author Contributions

H-JW and W-YL conceived the project and provided the funding. H-JW and W-YL wrote the manuscript. H-JW, S-JW, and W-YL performed the experiments. W-YL and H-JW supervised the study. All the authors contributed to the article and approved the submitted version.

## Conflict of Interest

The authors declare that the research was conducted in the absence of any commercial or financial relationships that could be construed as a potential conflict of interest.
