# Supplementary figures and images for: Non-canonical Interaction Between O-Linked N-Acetylglucosamine Transferase and miR-146a-5p Aggravates High Glucose-Induced Endothelial Inflammation
Source: Front Physiol. 2020 Oct 30;11:1091. doi: 10.3389/fphys.2020.01091 (PMC7662465; doi:10.3389/fphys.2020.01091)

S\_Fig.1A

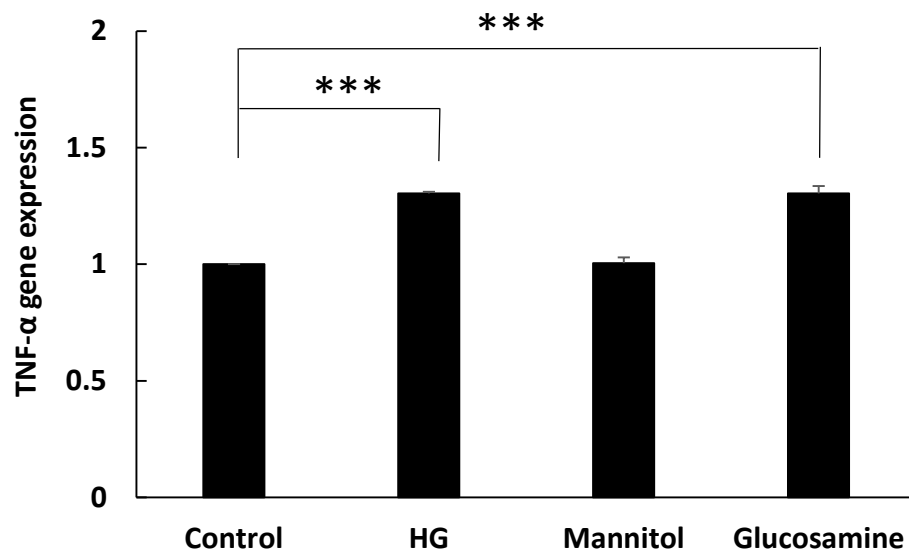

S\_Fig.1B

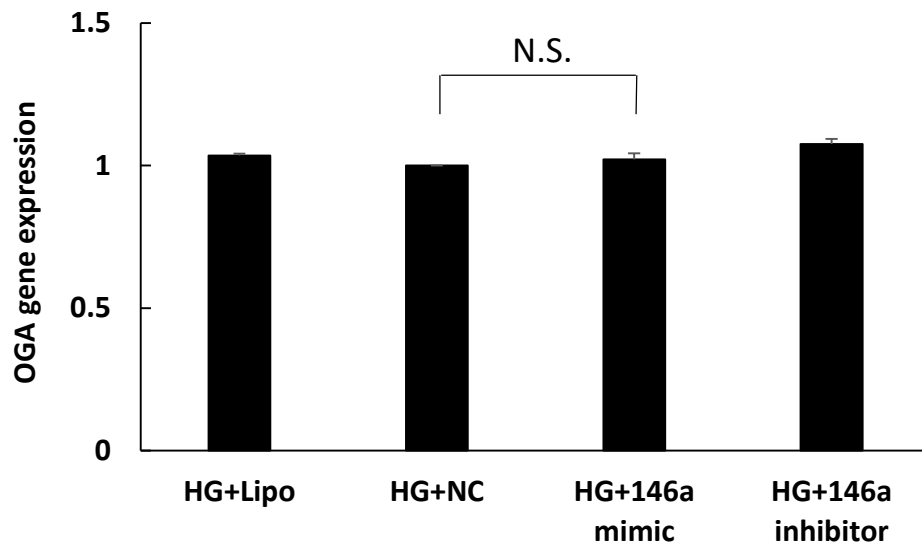

S\_Fig.1C

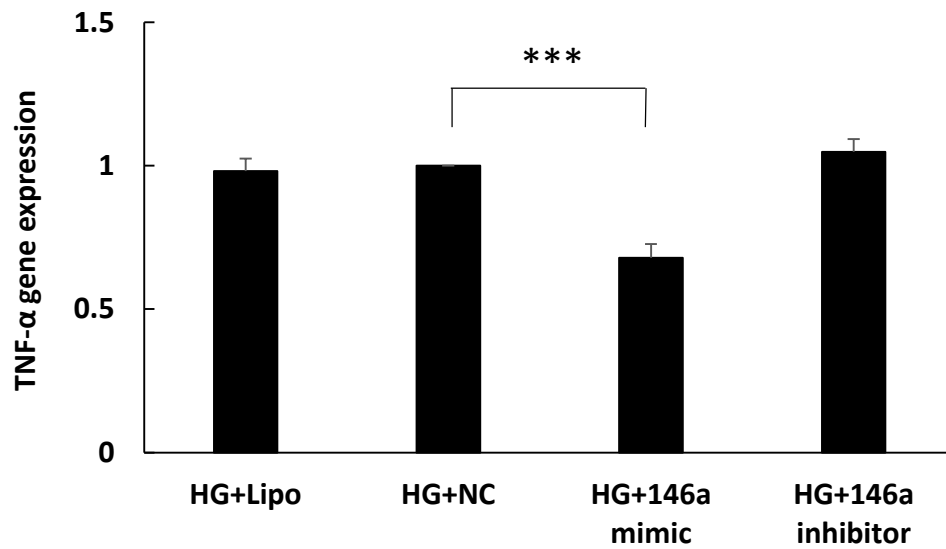

Supplement: Supplementary file 1 [file Data_Sheet_1.PDF]
